# *Cysticercus cellulosae* Regulates T-Cell Responses and Interacts With the Host Immune System by Excreting and Secreting Antigens

**DOI:** 10.3389/fcimb.2021.728222

**Published:** 2021-09-03

**Authors:** Xianmin Fan, Yue Zhang, Renhui Ouyang, Bo Luo, Lizhu Li, Wei He, Meichen Liu, Nan Jiang, Fengjiao Yang, Lingjun Wang, Biying Zhou

**Affiliations:** Department of Parasitology, School of Basic Medical Sciences, Zunyi Medical University, Zunyi, China

**Keywords:** *Taenia solium*, *Cysticercus cellulosae*, excretory secretory antigens, cellular immune response, T cells

## Abstract

*Cysticercus cellulosae* (*C. cellulosae*) excretes and secretes antigens during the parasitic process to regulate the host immune response; however, resulting immune response and cytokine production in the host during infection still remains unclear. We used *C. cellulosae* crude antigens (CAs) as controls to explore the effect of excretory secretory antigens (ESAs) on T-cell immune responses in piglets. *C. cellulosae* ESAs induced imbalanced CD4^+^/CD8^+^ T-cell proportions, increased the CD4^+^Foxp3^+^ and CD8^+^Foxp3^+^ T-cell frequencies, and induced lymphocytes to produce interleukin-10, which was mainly attributed to CD4^+^ and CD4^−^CD8^−^ T cells. The ESAs also induced Th2-type immune responses. The results showed that the ability of *C. cellulosae* to escape the host immune attacks and establish a persistent infection may be related to host immune response regulation by the ESAs.

## Introduction

*Taenia solium* (*T. solium*) is a cestode parasite that has zoonotic importance and is harmful to human health. Its developmental stages include four life cycles: eggs, oncospheres, larvae, and adults (Arora et al., 2017). Both larval form of this parasite, termed as *Cysticercus cellulosae* and the adult worms, are responsible for human disease. Adult parasites of *T. solium* infected the human intestine and caused taeniasis, whereas, *C. cellulosae* infected the subcutaneous tissues, muscle, brain, eyes, and other sites of intermediate host and caused cysticercosis, and neurocysticercosis (NCC) is the most serious form of the disease (Gonzales et al., 2016). Patients with NCC exhibit varying symptoms; these can be asymptomatic or have mild clinical manifestations (e.g., headache, dizziness, and occasional seizures) to a severe neurological syndrome (e.g., seizures, intracranial hypertension, hydrocephalus, cerebrospinal fluid blockage, cognitive deficit, and other features), which is chronic with a very high mortality rate (Fleury et al., 2016; Gonzales et al., 2016).

Parasitic infections are an important public health problem worldwide (Gazzinelli-Guimaraes and Nutman, 2018). Helminth parasites can regulate and evade host immune attacks through various means (Maizels and McSorley, 2016). Regulatory T cells (Tregs) could produce interleukin (IL)-10 and transforming growth factor (TGF)-β to suppress host immune responses (Arce-Sillas et al., 2016); thus, immune escape by parasites may be attributed to Tregs production. Host immunity determines parasite susceptibility (Tharmalingam et al., 2016), and CD4^+^ T cells play important roles in host immunoregulation (Zhu et al., 2010). Dendritic cells (DCs) are the most efficient antigen-presenting cells (Clotilde and Amigorena, 2001), which can present foreign antigens to naive CD4^+^ T cells, which then activate and differentiate into Tregs and helper T cells (Th), including Th1, Th2, and Th17. Th1-type immune responses inhibit infections, and Th2-type immune responses aggravate infections (Tharmalingam et al., 2016).

Both larval and adult form of *T. solium* excrete and secrete various products into the host; these products make direct contact with the hosts’ immune system and interfere with immunoregulation (Hewitson et al., 2009; White and Artavanis-Tsakonas, 2012; Sun et al., 2019). Among these products, excretory secretory antigens (ESAs) include many antigenic substances, which can be obtained *in vitro via* cultivation technology (Iddawela et al., 2007; Stefan et al., 2011; Wang et al., 2017). Compared with parasitic crude antigens (CAs) and soluble antigens (SAs), ESAs are involved in parasitic pathogenesis and immune evasion and are considered diagnostic and treatment targets (Hayam et al., 2013; Gazzinelli-Guimaraes and Nutman, 2018). For example, CD4^+^ and CD8^+^ T-cell proliferation are decreased and the mice’s immunoreaction to the worms is suppressed when mice are infected with ESAs from adult *hookworms*, thus increasing the worm’s ability to survive in the host (Nicholas and Blaise, 2017). ESAs from adult *nematodes* can selectively inhibit interferon (IFN)-γ and IL-2 production, but not IL-4 or IL-10 production, in CD4^+^ and CD8^+^ T cells of rat mesenteric lymph nodes. It shows that Th2 immune responses are closely related to persistent parasitic infections (Uchikawa et al., 2000).

*C. cellulosae* ESAs from *T. solium* induced angiogenesis in the brains of rat models of NCC, which may be related to the NCC pathology (Carmen-Orozco et al., 2019). Studies have shown that the sensitivity and specificity of enzyme-linked immunoelectrotransfer blots (EITB) for detecting anti-ESA antibodies in the serum of patients with NCC were higher than the lower molecular mass (LMM) antigenic fractions of *C. cellulosae*. Moreover, detecting anti-ESA antibodies *via* enzyme-linked immunosorbent assay (ELISA) enables evaluating the treatment responses of these patients. *C. cellulosae* survival is directly related to the existence of anti-ESA antibodies, and these antibodies can be used in new diagnostic tests and subsequent treatments (Molinari et al., 2002;Atluri et al., 2010; Atluri et al., 2014; Paredes et al., 2016). Thus, we conducted this study to explore the effects of *C. cellulosae* ESAs from *T. solium* on the immunoresponses of T lymphocytes.

## Materials and Methods

### Animals and Infection

A whole adult *T. solium* worm was obtained from a patient with taeniasis from a taeniasis-endemic area in Yajiang, Ganzi, Sichuan Province. To generate *T. solium* larvae, each healthy piglet was infected by feeding 5 pieces mature gravid proglottids from the worm at the Animal Experimental Center of Zunyi Medical University. There were 12 piglets in this infection experiment, including 10 piglets in the infection group and 2 piglets in the control group. The healthy piglets were confirmed to be pathogen-free before infection and raised under standard conditions. The Animal Care and Use Committee of Zunyi Medical University approved the experiments.

### Preparation of *C. cellulosae* ESAs and CAs

To prepare the ESAs and CAs of the *T. solium* larvae, the infected piglets were euthanized 2–3 months postinfection, and *C. cellulosae* were harvested from the muscle tissue. The *C. cellulosae* were washed three times in physiological saline and sterile phosphate-buffered saline (PBS: 0.02 M Na_2_HPO_4_/NaH_2_PO_4_, 0.15 M NaCl, pH 7.2), then cultivated at 37°C and 5% CO_2_ in RPMI 1640 medium (cat. #31800, Solarbio, Beijing, China) containing penicillin G (100 U/ml) and streptomycin (100 μg/ml). Then the culture supernatants were collected aseptically after 72 h. The supernatants were filtered through 0.22 μm sterile filters, then concentrated by transferring them into a 3 kDa ultrafiltration tube (cat. #UFC900396, Millipore, MA, USA). The liquid after concentration was *C. cellulosae* ESAs, and its protein concentration was determined using a Bradford protein assay kit (cat. #UFC0010, Solarbio, Beijing, China) per the manufacturer’s instructions and stored at −80°C until used.

The *C. cellulosae* CAs were prepared as previously described with some modifications (Verastegui et al., 2001). The *C. cellulosae* were collected and washed three times with physiological saline and PBS, then homogenized in a homogenizer (70 Hz/s, 10 s/time) at 4°C until completely lysed. The supernatants were collected *via* centrifugation as *C. cellulosae* CAs, and its protein concentration was measured using a Bradford protein assay kit per the manufacturer’s instructions and stored at −80°C until further use.

### Peripheral Blood Mononuclear Cell Isolation

Peripheral blood mononuclear cells (PBMCs) were isolated as previously described with minor modifications (Valent et al., 2017). Briefly, under sterile conditions, 10 ml of heparinized anticoagulated blood was collected from the healthy piglets *via* jugular venipuncture and diluted 1:1 with sample diluent according to the lymphocyte separation solution kit (cat. #LTS1110, TBD Sciences, Tianjin, China) as per manufacturer’s instructions. The diluted blood was added to the surface of an equal volume of separation liquid. The solutions were centrifuged at 2,500 rpm for 20 min according to the density gradient centrifugation to obtain the PBMCs layer. PBMCs were collected and washed twice with cleaning solution. Erythrocyte lysis buffer (cat. #R1010, Solarbio, Beijing, China) was used to eliminate erythrocyte contamination. PBMCs were suspended in RPMI 1640 medium with 10% inactivated fetal calf serum (cat. #11011-8611, Sijiqing, Zhejiang, China) after ensuring that the cell viability exceeded 95% *via* trypan blue staining.

### Cell Cultures

To analyze the effects of ESAs and CAs on mitogen-induced lymphocyte proliferation, PBMCs were cultured in 96-well plates at 1×10^6^ cells/well at 37°C and 5% CO_2_ and separately incubated with control medium, concanavalin A (ConA, 10 μg/ml) (cat. #C5275, Sigma, Saint Louis, MO, USA), ESAs (40 μg/ml), or CAs (40 μg/ml). Phytohemagglutinin (cat. #P8090, Solarbio, Beijing, China) was added to a final concentration of 2.5 μg/ml after cultivation for 3 h. Cells were harvested on day 2, stained with CD4 and CD8, and analyzed *via* flow cytometry.

To further analyze whether ESAs and CAs can induce Treg production, PBMCs were incubated in 96-well plates at 1×10^6^ cells/well and treated with control medium, ConA, ESAs, or CAs for 48 h under standard conditions. The cells were then collected and analyzed for Foxp3, CD4, and CD8 expression by flow cytometry. Foxp3 and Helios mRNA expression levels were calculated *via* quantitative real-time PCR (qRT-PCR).

To investigate whether ESAs and CAs can induce IL-10 production, PBMCs were cultured in 96-well plates at 1×10^6^/well and added with ESAs, CAs, 2 μg/ml lipopolysaccharide (LPS: cat. #L2880, Solarbio, Beijing, China), ESA+LPS, CA+LPS, or control medium. The culture supernatants were harvested after 24 h, and IL-10 expression was measured *via* ELISA.

The lymphocyte subsets expressing IL-10 were evaluated *via* intracellular cytokine staining. PBMCs were cultured in 96-well plates at 1×10^6^/well and stimulated with ESAs, CAs, LPS, ESA+LPS, CA+LPS, or control medium. During the last 12 h of culturing, 5 μg/ml of Brefeldin A (cat. #abs810012, Absin, Shanghai, China) was added to block intracellular cytokine secretion into the extracellular area. After 24 h, the cells were stained with IL-10, CD4, CD8, IL-10, and Foxp3 and analyzed *via* flow cytometry.

### T-Lymphocyte Polarization

Healthy piglets were anesthetized, 20 ml bone marrow was extracted, and the bone marrow precursor cells were isolated under sterile conditions. The cells were cultured under standard conditions, and the immature DCs were induced. The purity of immature DCs was more than 80% by flow cytometry. To determine the influence of *C. cellulosae* ESAs and CAs on T-cell polarization, CD4^+^ T cells were enriched from the spleens of the healthy piglets *via* positive selection using anti-CD4 magnetic beads (cat. #130-091-652, Miltenyi Biotec, Bergisch Gladbach, Germany) and a lymphocyte separation solution kit (cat. #LTS1110, TBD Sciences, Tianjin, China) as per manufacturer’s instructions. CD4^+^ T cells at 1×10^7^ were co-cultivated with 1×10^6^ immature DCs in 96-well pates and added with ESAs (40 μg/ml), CAs (40 μg/ml), ConA (10 μg/ml), or control medium stimulation. The medium was removed *via* centrifugation, and 10 ng/ml pig recombinant IL-2 (cat. #ab238302, ABCAM, Cambridge, UK) was added to promote polarization and maintain T-lymphocyte activity when 24 h of culture. After 48 h, the culture supernatants were collected, and the expression levels of IFN-γ (Th1), IL-4 (Th2), and IL-17 (Th17) were analyzed *via* ELISA.

### Flow Cytometry and Antibodies

For cell surface staining, cells were washed twice in precooling staining buffer, then resuspended in staining buffer and incubated with primary antibodies for 30 min on ice in the dark. Cells were then washed once with staining buffer and resuspended in it. The antibodies were used as CD4a:PE-Cy7 (clone #561473, BD Pharmingen, Franklin Lakes, NJ, USA) and CD8a:AF647 (clone #561475, BD Pharmingen, Franklin Lakes, NJ, USA). To detect the intracellular antigens, cells were first incubated for 30 min with surface-labeled antibodies, then washed and resuspended in staining buffer. Cells were then fixed and permeabilized for 45 min with transcription factor buffer (cat. #562574, BD Pharmingen, Franklin Lakes, NJ, USA) as per manufacturer’s instructions, then incubated with intracellular antigens fluorescent antibodies for 45 min on ice in the dark. The antibodies were used as CD4a:PE-Cy7, CD8a:AF647, Foxp3:FITC (clone #11577382, Thermo Fisher, Waltham, MA, USA), and IL-10:PE (clone #12710841, Thermo Fisher, Waltham, MA, USA).

### ELISA for Cytokine Secretion

Cytokine secretion was detected using pig IFN-γ, IL-4, and IL-17 ELISA kits (cat. #SEA056Po, Cloud-Clone Corp., Wuhan, China). The culture supernatants were collected, and then the cytokine production levels were analyzed as per manufacturer’s instructions.

### qRT-PCR

The relative changes of Foxp3 and Helios mRNA expression levels in the PBMCs were measured *via* qRT-PCR. The primer sequences used were pig GAPDH (F: 5′-TCGGAGTGAACGGATTTGGC-3′, R: 5′-TGACAAGCTTCCCGTTCTCC-3′), pig Foxp3 (F: 5′-GGTGCAGTCTCTGGAACCAAC-3′, R: 5′-GGTGCCAGTGGCTACAATAC-3′), and pig Helios (F: 5′-AGGAGGTACATGGTGACTCA-3′, R: 5′-CTCACAGGACACCTCAGGAC-3′). TRIzol universal total RNA extraction reagent (cat. #D424, Tiangen) was used to extract total RNA from the stimulated cells per the manufacturer’s instructions. Reverse transcription and qRT-PCR were performed using RevertAid First Strand cDNA synthesis kit (cat. #K1622, Thermo Fisher) and FastStart Universal SYBR Green Master (Rox) (cat. #04 913 914 001, Roche, Basel, Switzerland). The cycle conditions were one cycle at 95°C for 15 s, 56°C for 30 s, and 72°C for 20 s for 44 cycles. The relative Foxp3 and Helios mRNA expression levels were determined *via* the 2^−ΔΔCT^ method.

### Statistical Analysis

One-way analysis of variance was used for multiple-group comparisons, and the least significant differences test was used for pairwise comparisons between groups. *P*<0.05 was considered the lowest statistically significant; *P*<0.01 was considered moderately statistically significant; P<0.001 was considered the highest statistically significant.

## Results

### *C. cellulosae* ESAs and CAs Induced CD4^+^ and CD8^+^ T-Lymphocyte Responses

**Figure 1:**
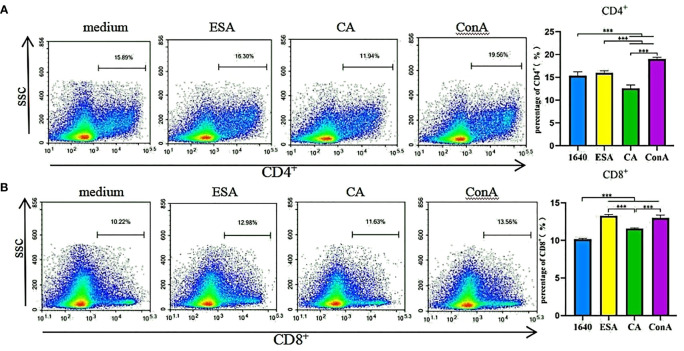
T cells immune response assay. The frequencies of CD4^+^ and CD8^+^ T lymphocytes were detected by flow cytometry. **(A)** *Cysticercus cellulosae* CA inhibited the expression of CD4^+^ T lymphocytes but ESA had no significant effect on them. *Cysticercus cellulosae* ESA significantly induced the expression of CD4^+^ T lymphocytes compared with CA. **(B)** Both *Cysticercus cellulosae* ESA and CA induced the production of CD8^+^ T lymphocytes, and compared with CA, ESA significantly stimulated the expression of CD8^+^ T lymphocytes. All dates were represented by means ± SD, ***P < 0.001.

Piglet PBMCs were cultured with ESAs and CAs for 48 h, then the CD4^+^ and CD8^+^ T-cell subset expressions were detected *via* flow cytometry. The ESAs-induced CD8^+^ T cells increased to levels near those of ConA. Compared with the normal controls, the CAs-induced CD8^+^ T cells were elevated, but CD4^+^ T cells were decreased, and both levels were lower than those of ConA (**Figure 1**). Compared with CAs, ESAs induced a significant increase in CD4^+^ and CD8^+^ T cells (**Figures 1A, B**). Thus, both *C. cellulosae* ESAs and CAs can stimulate an immune imbalance in the T-lymphocyte subsets.

### *C. cellulosae* ESAs and CAs Stimulated Tregs Subsets Expression

**Figure 2:**
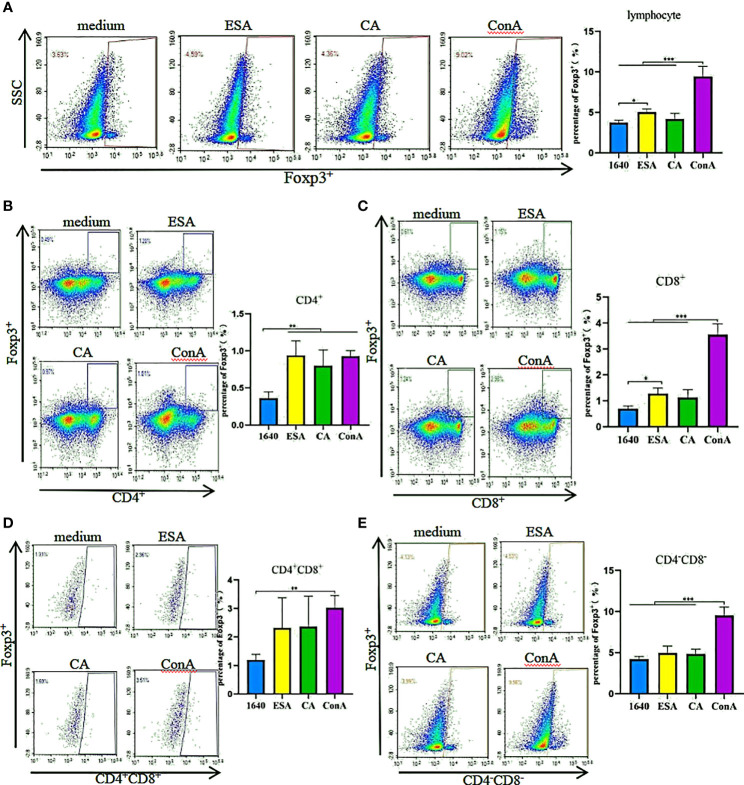
Tregs inducing assay. PBMCs were stimulated for 48 hours with *Cysticercus cellulosae* ESA and CA respectively, then the cells were collected and measured the expression of Tregs subsets via flow cytometry. **(A)** *Cysticercus cellulosae* ESA significantly induced the expression of Foxp3^+^ lymphocytes, while, CA had no obvious impacts on the expression. **(B)** Both *Cysticercus cellulosae* ESA and CA could promote the frequency of CD4^+^Foxp3^+^Tregs. **(C)** The frequency of CD8^+^Foxp3^+^ Tregs was increased only by *Cysticercus cellilosae* ESA. **(D, E)** Both *Cysticercus cellulosae* ESA and CA had no effects on the expression of CD4^+^CD8^+^Foxp3^+^ and CD4^-^CD8^-^Foxp3^+^Tregs. Dates were expressed as the means ± SD, *P < 0.05, **P < 0.001, ***P < 0.001.

The effects of *C. cellulosae* ESAs and CAs on Treg production were checked. We analyzed the expressions of Treg subsets *via* flow cytometry. *C. cellulosae* ESAs increased Foxp3^+^ lymphocyte expression and had a statistical significance. The CAs also slightly increased the Foxp3^+^ lymphocytes, but the increase was not statistically significant compared with the controls (**Figure 2A**). Tricolor flow cytometry showed that *C. cellulosae* ESAs stimulated CD4^+^ and CD8^+^ lymphocytes, which are the main source of Foxp3 expression. CAs stimulated only the production of CD4^+^Foxp3^+^ Tregs (**Figures 2B, C**). Neither the ESAs nor the CAs of *C. cellulosae* significantly affected the expressions of CD4^+^CD8^+^Foxp3^+^ or CD4^−^CD8^−^Foxp3^+^ Tregs (**Figures 2D, E**).

### *C. cellulosae* ESAs and CAs Decreased the Relative *Foxp3* and *Helios* mRNA Expression Levels

**Figure 3:**
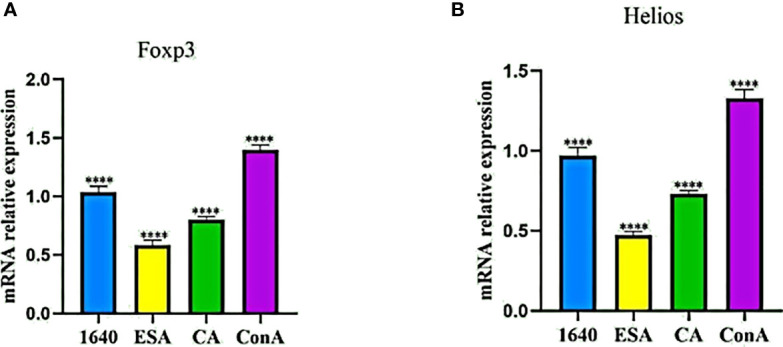
qRT-PCR revealed the mRNA expression levels of Foxp3 and Helios in PBMCs. **(A)** The Foxp3 mRNA expression quantity was inhibited by *Cysticercus cellulosae* ESA and CA compered with 1640 group. **(B)** Similiar to the change of Foxp3, ESA and CA also reduced the mRNA expression level of Helios inPBMCs. Results were expressed as the mean ± SD, ****P < 0.0001.

Because Foxp3^+^ Tregs also express Helios, we preliminarily detected the mRNA expression levels of Foxp3 and Helios in PBMCs stimulated with *C. cellulosae* ESAs and CAs *via* qRT-PCR. Compared with those of the normal controls, *C. cellulosae* ESAs and CAs significantly reduced the Foxp3 and Helios mRNA expression levels in the PBMCs (**Figures 3A, B**).

### IL-10 Secretion in PBMCs

**Figure 4:**
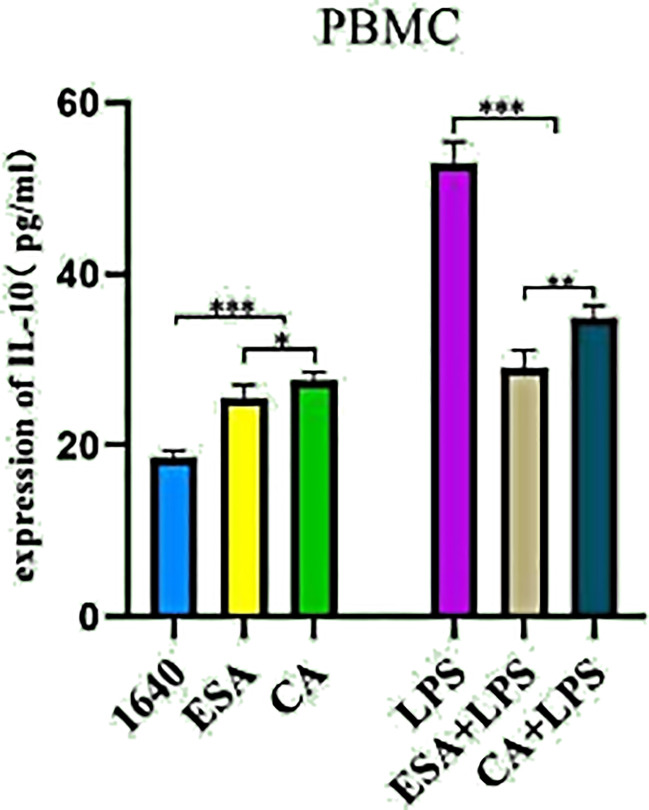
IL-10 secretion. The secretion level of IL-10 in cultured supernatant was measured by ELISA. *Cysticercus cellulosae* ESA and CA treated PBMCs for 24 hours without LPS, ELISA showed both ESA and CA could be capable to induce IL-10 secretion. But, under the presence of LPS, the secretion of IL-10 was inhibited by ESA and CA in PBMCs. Dates were represented by means ± SD, *P < 0.05, **P < 0.01, ***P < 0.001.

PBMCs were stimulated with ESAs and CAs for 24 h, then the culture supernatants were collected, and the IL-10 secretion levels were detected *via* ELISA. In the absence of LPS, both *C. cellulosae* ESAs and CAs increased the IL-10 secretion in the PBMCs compared with that of the controls. Additionally, *C. cellulosae* ESAs induced lower IL-10 secretion levels than did the CAs. However, stimulating the ESAs and CAs with LPS significantly reduced the IL-10 secretion levels. Compared with CAs+LPS, ESAs+LPS decreased the IL-10 secretion (**Figure 4**).

### *C. cellulosae* ESAs and CAs Induced IL-10 Expression in Lymphocytes

**Figure 5:**
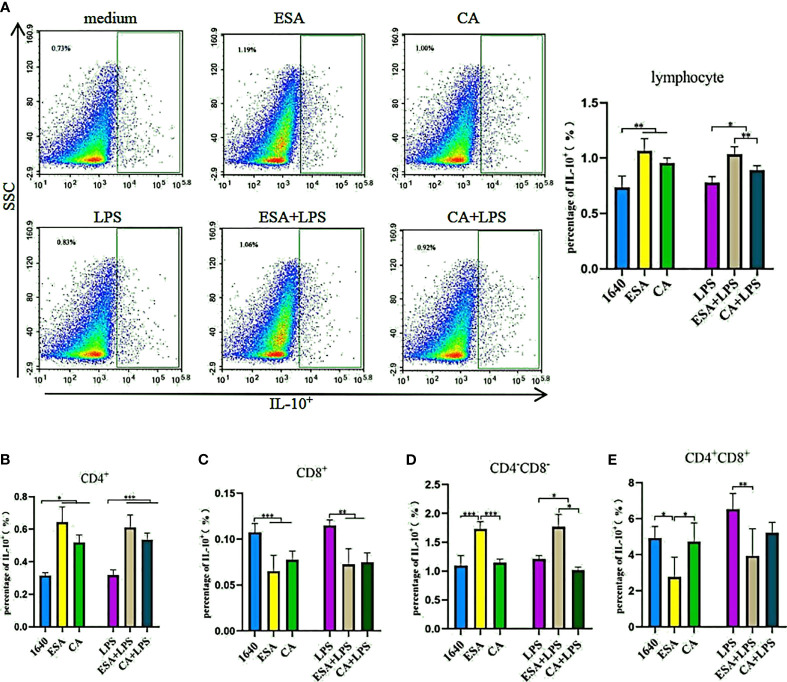
IL-10^+^ lymphocyte assay. The production of IL-10^+^ lymphocyte subsets by flow cytometry. **(A)** Both *Cysticercus cellulosae* ESA and CA induced the production of IL-10^+^ lymphocyte. **(B)** CD4^+^T lymphocyte was the main source *Cysticercus cellulosae* ESA and CA-induced IL-10 expression. **(C)** In the presence or absence of LPS, *Cysticercus cellulosae* ESA and CA significantly inhibited the expression frequency of CD8^+^IL-10^+^ lymphocyte. **(D)** *Cysticercus cellulosae* ESA could be induced the expression of CD4^-^CD8^-^IL-10^+^ cell under the presence or absence of LPS. While, CA only decreased the expression in the presence of LPS. **(E)** Similar to **(C)**, the production of IL-10 in CD4^+^CD8^+^ lymphocyte was inhibited by *Cysticercus cellulosae* ESA. All dates were shown as the means ± SD, *P < 0.05, **P < 0.01, ***P < 0.001.

To further investigate IL-10 expression in lymphocytes, we used tricolor flow cytometry to detect the IL-10^+^, CD4^+^IL-10^+^, CD8^+^IL-10^+^, and CD4^+/−^CD8^+/−^IL-10^+^ lymphocyte frequencies. Without LPS, both *C. cellulosae* ESAs and CAs significantly increased the IL-10^+^ lymphocyte frequencies. Additionally, the induction effects of the ESAs and CAs on IL-10^+^ lymphocytes were stronger in the presence LPS (**Figure 5A**). CD4^+^ T lymphocytes were the main sources of *C. cellulosae* ESA- and CA-induced IL-10 secretion (**Figure 5B**). ESAs also induced CD4^−^CD8^−^IL-10^+^ lymphocyte expression (**Figure 5D**). Conversely, IL-10 expression was significantly inhibited in the CD8^+^ and CD4^+^CD8^+^ T lymphocytes (**Figures 5C–E**). When cells were co-cultured with *C. cellulosae* ESA+LPS and CA+LPS, the CD4^+^IL-10^+^ lymphocyte frequencies increased markedly, but the CD8^+^IL-10^+^ lymphocyte expression decreased significantly (**Figures 5B, C**). *C. cellulosae* ESAs stimulated with LPS significantly inhibited the frequency of CD4^+^CD8^+^IL-10^+^ cells but increased the expression frequency of CD4^−^CD8^−^IL-10^+^ cells. However, *C. cellulosae* CAs stimulated with LPS decreased the CD4^−^CD8^−^IL-10^+^ cells production (**Figures 5D, E**).

**Figure 6:**
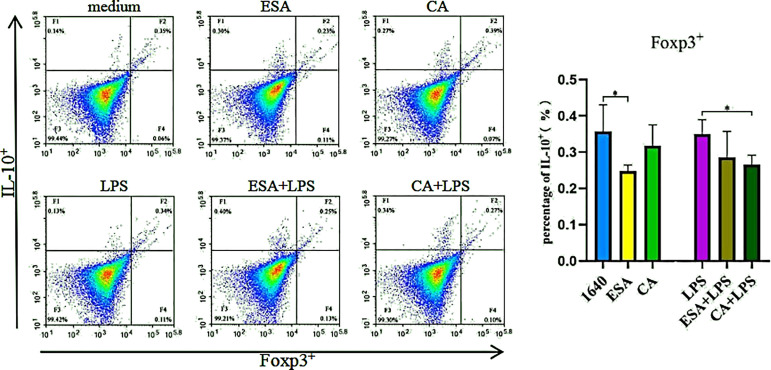
The production of Foxp3^+^IL-10^+^lymphocyte. The co-expressing Foxp3 and IL-10 cell were detected *via* tricolor flow cytometry, which shown that the production of Foxp3^+^IL-10^+^ lymphocyte was inhibited by *Cysticercus cellulosae* ESA. Besides, CA had significantly decreased the production only under the presence of LPS. Results were expressed as the mean± SD, *P < 0.05.

Co-staining IL-10 and Foxp3 without LPS revealed that *C. cellulosae* ESAs inhibited the Foxp3^+^IL-10^+^ lymphocyte production, but no significant differences were noted between the CAs and the controls. Interestingly, only *C. cellulosae* CAs reduced the Foxp3^+^IL-10^+^ lymphocytes expression when combined with LPS (**Figure 6**).

### Th1, Th2, and Th17 Cell Differentiation

**Figure 7:**
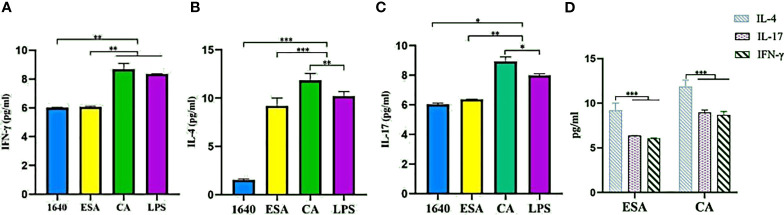
Th cytokines secretion. The secretion levels of IFN-γ, IL-4 and IL-17 were measured by ELISA. **(A)** Only *Cysticercus cellulosae* CA induced IFN-γ secretion, ESA had no effect on it. **(B)** Both *Cysticercus cellulosea* ESA and CA significantly induced the production of IL-4 and the latter was higher than the former. **(C)** *Cysticercus cellulosae* CA obviously up-regulated the secretion level of IL-17 compared with ESA. **(D)** Both *Cysticercus cellulosae* ESA and CA dramatically induced IL-4 secretion. All dates were represented by means ± SD, *P < 0.05, **P < 0.01, ***P < 0.001.

To investigate the modulatory effects of *C. cellulosae* ESAs and CAs on Th subset production, IFN-γ, IL-4, and IL-17 secretion levels were examined *via* ELISA. DC-CD4^+^ T cells exposed to *C. cellulosae* ESAs and CAs produced different cytokine levels (**Figures 7A–C**). Stimulation with *C. cellulosae* CAs significantly induced IL-4, IL-17, and IFN-γ secretion, and the IL-4 and IL-17 secretion levels were higher than those of the positive controls. However, *C. cellulosae* ESAs strongly promoted IL-4 and IL-17 secretion, but it was lower than that of the CAs. Importantly, both ESAs and CAs dramatically induced IL-4 production (**Figure 7D**). Therefore, *C. cellulosae* ESAs and CAs stimulation played strong regulatory roles in Th cell immune responses by altering the cytokine secretion.

## Discussion

Cysticercosis is a complex disease involving host-parasite interactions. The extent and nature of the host immune response, as well as the active immunomodulatory mechanisms produced by the parasite, determine the disease occurrence and development (Garcia et al., 2014). Under normal conditions, T-cell subsets regulate each other and maintain a balanced immune function. In this study, *C. cellulosae* ESAs and CAs increased the number of CD8^+^ T cells without significantly changing or decreasing the number of CD4^+^ T cells, thus decreasing the CD4^+^/CD8^+^ T-cell ratios. Thus, the immune imbalance induced by cysticercosis may be related to regulation of the T-cell immune responses by *C. cellulosae* ESAs and CAs, indicating that these two have potential regulatory roles in lymphocytes homeostasis.

Similar to human Tregs, CD4^+^Foxp3^+^ T cells are markers of porcine Tregs, and porcine CD8^+^Foxp3^+^ Tregs have also been identified (Wang et al., 2016). Tregs can exert suppressive functions by prompting secretion of the immunosuppressive cytokines, IL-10 and TGF-β, as well as play important roles in immunotolerance and immune homeostasis (Georgiev et al., 2019). The transcription factor, Foxp3, a dominant regulator and highly specific marker of Tregs, is the prerequisite for Treg cells to produce and exert their immunosuppressive effects (Deng et al., 2019). Foxp3-defective Tregs have decreased IL-10 and TGF-β production levels and weaker suppression abilities (Jia et al., 2015). During parasitic infections, Tregs are increased and exert immunosuppressive functions, enabling the parasites to escape immune attack and survive for long periods in the host. Here, *C. cellulosae* ESAs induced Foxp3 expression in piglet lymphocytes, but the CAs showed no significant differences compared with those of the controls. These results are similar to those of a previous study on *Toxocara canis* ESAs in canine Foxp3^+^ Tregs (Junginger et al., 2017). The results showed that *C. cellulosae* ESAs may induce Treg production and exert immunosuppressive effects in natural hosts. Further analysis of the lymphocyte subsets expressing Foxp3 showed that ESAs and CAs promoted Foxp3 expression in CD4^+^T lymphocytes, which are typical immunosuppressive cells. Additionally, *C. cellulosae* ESAs also induced production of CD8^+^Foxp3^+^ Tregs, which inhibited T-cell activation and proliferation and induced *de novo* generation of CD4^+^Foxp3^+^ Tregs (Erika et al., 2012). These results were similar to those of previous studies in that induction of Tregs by parasitic ESAs was more significant than that of CAs (Wang et al., 2015; Grainger et al., 2020). The differences in Treg subsets induced by ESAs and CAs may be related to the different components of the two antigens. In summary, ESAs and CAs could induce Treg expression, which may be one mechanism by which *C. cellulosae* evades the host immune attacks.

Foxp3^+^ Tregs can be divided into Helios^+^ and Helios^−^ Tregs in mice. In human Tregs, Helios knockout downregulated Foxp3 expression, thereby weakening Treg inhibition (Eyad et al., 2015). qRT-PCR was used to detect relative mRNA expression levels of Foxp3 and Helios in ESA- and CA-stimulated cells to preliminarily evaluate the change trend of Foxp3 and Helios in these cells. The relative expression trend of the Helios mRNA coincided well with the Foxp3 mRNA in PBMCs stimulated with *T. solium* larva ESAs and CAs and were significantly lower than those of the controls. One study found reduced Foxp3 mRNA levels in splenocytes and placental cells in mice infected with *Toxoplasma gondii* (Ge et al., 2010). Helios was originally thought to be expressed only in Tregs, but studies have shown that it is also expressed in CD8^+^ cells, NK cells, B cells, and other human and mouse cells, but its function in these cells is unclear (Eyad et al., 2015; Thornton and Shevach, 2019). In summary, whether porcine Foxp3 and Helios are expressed in immune cells other than CD4^+^ and CD8^+^ T cells and whether play immunosuppressive roles in these immune cells require further study.

IL-10 is an inhibitory anti-inflammatory factor that inhibits T-cell proliferation by inhibiting the production of proinflammatory factors and downregulating the expression of MHC-II and costimulatory molecules on the surface of antigen-presenting cells and directly acts on T cells to induce an unresponsive state (Kumar et al., 2019; Ouyang and O’Garra, 2019; Saraiva et al., 2019). Clinical malaria models and human studies have shown that IL-10 could inhibit antiparasitic immunity (Kumar et al., 2019). In our study, *C. cellulosae* ESAs and CAs stimulated IL-10 secretion in PBMCs, which is similar to the findings of previous (Javaid et al., 2016). In summary, increased IL-10 secretion may inhibit the host’s immune responses against cysticercosis, leading to a persistent infection. One study showed that LPS induced IL-10 synthesis and release (Chanteux et al., 2007). In addition to Treg cells, IL-10 is also derived from Th2, CD4, and CD8 T lymphocytes (Weiss et al., 2011). Additionally, *C. cellulosae* ESAs and CAs significantly inhibited IL-10 secretion in the presence of LPS. This result may be related to the interregulation of IL-10 among different cells.

Three-color flow cytometry showed that CD4^+^ T lymphocytes were mainly responsible for ESA- and CA-induced IL-10 expression. Their production can inhibit nitrous oxide pathway-mediated insecticidal immunity and protect parasites from host immune attacks (Zhou et al., 2014). Additionally, ESAs can also induce production of CD4^−^CD8^−^IL-10^+^ T lymphocytes, which can express IL-10 *in vitro* and inhibit CD4^+^ T-cell proliferation (Passos et al., 2017). Thus, CD4^+^IL-10^+^ and CD4^−^CD8^−^L-10^+^ T cells play essential roles in immunoregulation of parasitic infections, which may be one mechanism of immune evasion by *C. cellulosae*. ESAs and CAs can also regulate the production of IL-10^+^ lymphocytes *via* LPS-related pathways, but the specific molecular mechanism remains unclear. Although ESAs and CAs can induce the IL-10^+^, CD4^+^IL-10^+^ and CD4^−^CD8^−^IL-10^+^T lymphocyte production, it is unknown whether these cells represent Tregs. Further found ESAs inhibited Foxp3^+^IL-10^+^ cell production, but CAs did not. In summary, IL-10 secretion in lymphocytes induced by *C. cellulosae* ESAs and CAs may be unrelated to Foxp3 expression; the two were likely independent of each other and exerted inhibitory effects.

Th cells specialize in producing effector cytokines and play significant roles in adaptive immune responses. Activated CD4^+^ T cells can obtain different effector phenotypes, such as those of Th1, Th2, and Th17 (Bianchi et al., 2000). IFN-γ produced by Th1 and IL-4 produced by Th2 can amplify themselves but antagonize each other (Wan and Flavell, 2009). Once infected, the pathogens will actively regulate the immune balance of Th1/Th2 cells and escape from host immune attacks. Both *C. cellulosae* CA and ESA stimulation led to imbalanced Th immune responses and dominated by Th2-type immunity, which could be interpreted as the manifestation of cytokine cross-regulation. Th2-type immune responses are related to parasite susceptibility (Mendlovic et al., 2015), which may be one reason for the persistent development of cysticercosis. *C. cellulosae* CAs induced Th2-type immune responses more significantly than did the ESAs, which may be related to the different abilities of DCs antigen presentation induced by the two antigens and the change in Th-type responses with the infection course. The specific immunoregulatory mechanism requires further study (Xu et al., 2016; Musumeci et al., 2019).

## Conclusion

In summary, both *C. cellulosae* ESAs and CAs induced T-cell immune imbalances, which in turn induced immunosuppressive Treg cells, IL-10, and Th2-type immune response production. These may be related to the ability of *C. cellulosae* to evade the host immune attacks. Our research provides an experimental basis for developing anticysticercosis treatments and disease control strategies, but the cellular and molecular mechanisms involved in regulation of the host immune responses by *C. cellulosae* require further study.

## Data Availability Statement

The raw data supporting the conclusions of this article will be made available by the authors, without undue reservation.

## Ethics Statement

The animal study was reviewed and approved by the Ethics Committee of Zunyi Medical University. Written informed consent was obtained from the owners for the participation of their animals in this study.

## Author Contributions

XF, YZ, RO, BL, and FY conducted the experiments. XF, YZ, and BZ designed the experiments. XF analyzed the data. LL, WH, ML, NJ, LW, and BZ assisted with the experiments. XF wrote the manuscript. BZ revised the manuscript. All authors contributed to the article and approved the submitted version.

## Funding

This work was funded by the National Natural Science Foundation of China (81960378).

## Conflict of Interest

The authors declare that the research was conducted in the absence of any commercial or financial relationships that could be construed as a potential conflict of interest.

## Publisher’s Note

All claims expressed in this article are solely those of the authors and do not necessarily represent those of their affiliated organizations, or those of the publisher, the editors and the reviewers. Any product that may be evaluated in this article, or claim that may be made by its manufacturer, is not guaranteed or endorsed by the publisher.
